# A miRNA Binding Site Single-Nucleotide Polymorphism in the 3′-UTR Region of the IL23R Gene Is Associated with Breast Cancer

**DOI:** 10.1371/journal.pone.0049823

**Published:** 2012-12-11

**Authors:** Lihong Wang, Wei Liu, Wei Jiang, Jing Lin, Yongdong Jiang, Bo Li, Da Pang

**Affiliations:** 1 Institute of Cancer Prevention and Treatment, Harbin Medical University, Harbin, China; 2 Department of Pathology, The First Affiliated Hospital of Harbin Medical University, Harbin, China; 3 College of Bioinformatics Science and Technology, Harbin Medical University, Harbin, China; 4 Department of Breast Surgery, The Third Affiliated Hospital of Harbin Medical University, Harbin, China; 5 Department of Immunology, Harbin Medical University, Harbin, China; Democritus University of Thrace, Greece

## Abstract

**Background:**

Research into the etiology of breast cancer has recently focused on the role of the immunity and inflammation. Interleukin-23 and its receptor (IL23R) guide T cells towards the Th17 phenotype. IL23R single nucleotide polymorphisms (SNPs) have been shown to be associated with digestive system cancers. To evaluate the influences of IL23R gene polymorphisms on the risk of sporadic breast cancer, a case-control study was conducted in Chinese Han women.

**Methodology and Principal Findings:**

We genotyped two tag SNPs (rs10889677 in the 3′-UTR region and nonsynonymous variants rs1884444 in exon 2) in IL23R gene of 491 breast cancer patients and 502 matched healthy controls. The genotypes were determined using the SNaPshot technique. The differences in the genotypic distribution between breast cancer patients and healthy controls were analyzed with the Chi-square test for trends. For rs10889677 in IL23R, the frequencies of the AA genotype and the A allele were statistical significant higher in breast cancer patients than in controls (P = 0.0084 and P = 0.0171, respectively), whereas the C allele was associated with an earlier age of breast cancer onset (50.6 years for AA, 48.7 years for AC and 46.0 years for CC (P = 0.0114)) in case-only study. The clinical features analysis demonstrated significant associations between rs1884444 in IL23R and human epidermal growth factor receptor 2 (Her-2) and tumor size status.

**Conclusions and Significance:**

Our results suggest that a miRNA binding site SNP in the 3′-UTR region of the IL23R gene may be associated with the risk of breast cancer and contribute to the early development of breast cancer in Chinese women.

## Introduction

Breast cancer is the most common malignancy in women worldwide and its rate is increasing in both developed and developing countries [1]. The etiology of breast cancer is complicated and not completely understood, but recent studies have focused on the roles of immunity and inflammation [2]. Recently, a subset of T-helper 17 (Th17) cells characterised by interleukin-17A (IL-17A) production was implicated as a critical mediator of inflammation and cancer [3]. Our previous study suggested that SNPs in IL-17A were associated with the risk of breast cancer [4].

Interleukin-23 (IL-23) is essential for maintaining the Th17 response and is characteristically associated with Th17 cell lineage differentiation [5], [6]. The IL-23/IL-17 axis, therefore, is one of the main cytokine axes driving the pathogenesis of cancers [7], [8]. The receptor for IL-23 is composed of interleukin-12 receptor β1 (IL-12Rβ1), a common subunit shared with IL-12R, and a unique IL-23 receptor (IL23R) [9]. Moreover, IL23R are expressed on a variety of cells,including activated T cells, monocytes, macrophages, natural killer cells and dendritic cells that secrete IL-17 [10], [11]. In some recent studies, the IL23R gene was proved to be a susceptibility gene for several cancers. For example, the C allele of rs10889677 and the G allele of rs1884444 in IL23R may contribute to oral and gastric cancer susceptibility, respectively [12], [13]. And Chu et al found IL23R rs6682925 TC/CC and rs1884444 TG/GG variant genotypes were associated with significantly increased risk of esophageal cancer [14]. But presently, no studies evaluated the association between IL23R SNPs and the breast cancer risk.

Based on the Chinese (CHB) genotype data in HapMap database and the Haploview software, we found two tag SNPs rs10889677 and rs1884444 in IL23R gene. Among them, rs10889677 located at the 3′-untranslated region (3′-UTR) which can be regulated by regulatory proteins and microRNAs (miRNAs). And a variant in the 3′-UTR region could affect the stability and translation of the mRNA and further influence the amount of receptor protein expressed by a cell. Recently, Antonie et al reported that persons with rs10889677 A allele in the 3′-UTR displayed enhanced levels of both mRNA and protein production of IL23R, which could be attributed to a loss of binding capacity for the miRNAs Let-7e and Let-7f [15]. The SNP rs1884444 located at codon 3 in exon 2 of IL23R resulted in an amino acid His to Gln change, and the signal peptide of IL23R was encoded in exon 2 [16]. According to the web-based SNP analysis tool, PupaSuite2, the T to G base change of rs1884444 may disrupt an exonic splicing enhancer, resulting in exon skipping, malformation or transcript alternative splicing [14]. Thus we performed a genotyping of the two SNPs in a case-control study of 491 breast cancer patients and 502 matched healthy controls in Chinese Han women.

## Results

### The Genotypic and allelic frequencies of IL23R and the risk of breast cancer

The genotypic and allelic frequencies of the IL23R polymorphisms in breast cancer patients and healthy controls are shown in Table 1. The two SNPs genotyped were in Hardy-Weinberg equilibrium (HWE) (*P*>0.05) in our case and control cohorts.

**Table 1:** Frequencies of genotypes and alleles of two IL23R SNPs in breast cancer patients and controls.

Reference SNP ID	Genotype/Allele	Frequency no. (%)		*P* value	Odds ratio (95% CI)
		Patients (*n* = 491)	Controls (*n* = 502)		
rs10889677	A/A	267 (54.38)	231 (46.02)	0.0084	1.40 (1.09–1.79)
	C/A	182 (37.07)	221 (44.02)	0.0256	0.75 (0.58–0.97)
	C/C	42 (8.55)	50 (9.96)	0.4448	
	A	716 (72.91)	683 (68.03)	0.0171^a^	1.27 (1.04–1.53)
	C	266 (27.09)	321 (31.97)		
rs1884444	T/T	219 (44.60)	204 (40.64)	0.2064	
	G/T	216 (43.99)	234 (46.61)	0.4067	
	G/G	56 (11.41)	64 (12.75)	0.5160	
	T	654 (66.60)	642 (63.94)	0.2142	
	G	328 (33.40)	362 (36.06)		

a *P* = 0.0365 after correcting the *P*-value for multiple testing with the Haploview program using 10,000 permutations.

Only the rs10889677 SNP, located at the 3′-UTR of the IL23R gene, was associated with the risk of breast cancer. The frequency of the AA genotype was statistical significant higher while the frequency of the AC genotype was lower in breast cancer patients than in controls (*P* = 0.0084 and *P* = 0.0256, respectively). And the frequency of the A allele was statistical significant higher in breast cancer patients than in controls (*P* = 0.0171). After correcting the *P*-value for multiple testing with the Haploview program using 10,000 permutations, significant association was also found for this SNP (*P* = 0.0365). However, for the rs1884444 SNP, the genotype and allele frequencies did not differ significantly between the cases and the controls (*P*>0.05). Consistently, there was no significant association between this SNP and the risk of breast cancer.

### Age at onset of breast cancer and IL23R SNPs

**Figure 1:**
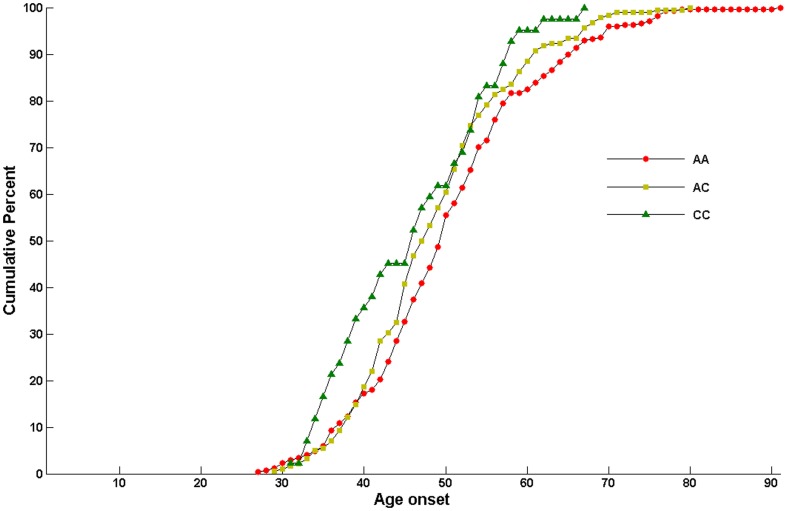
Age at onset of breast cancer and variants of IL23R gene.

Further case-only analysis revealed an association between rs10889677 genotype and mean age of diagnosis of breast cancer (i.e., a younger age of onset with increasing number of C alleles): 50.6±11.0 years (range, 27–91 years) for AA, 48.7±9.4 years (range, 29–80 years) for AC, and 46.0±9.3 years (range, 31–67 years) for CC. The carriers of rs10889677 AC or CC genotypes were 1.9 years or 4.6 years younger than that of AA genotypes (ANOVA test *P* = 0.0114) (Figure 1).

### Haplotypes analysis

The association of IL23R SNPs with breast cancer was further analyzed with the Haploview program. The frequency of the haplotype T_rs1884444_A_rs10889677_ was higher in patients than in controls (*P* = 0.0062), whereas the frequency of the haplotype T_rs1884444_C_rs10889677_ was lower (*P* = 0.0093) in patients than in controls (Table 2). After the *P*-value was corrected for multiple testing using the permutation option in the Haploview software, significant associations were found for the two haplotypes (*P* = 0.0179 and *P* = 0.0259, respectively).

**Table 2:** Haplotype frequencies in breast cancer patients and healthy controls.

Haplotype	Freq.	Breast Cancer (*n* = 491)	Healthy Controls (*n* = 502)	*P* value
TA	0.556	0.576	0.528	0.0062^a^
GC	0.199	0.188	0.207	0.4232
GA	0.149	0.140	0.155	0.4447
TC	0.097	0.078	0.114	0.0093^b^

The haplotypes shown occurred with a frequency of ≥10% in the case-control population.

a *P* = 0.0179 and

b *P* = 0.0259 after correcting the *P*-value for multiple testing with the Haploview program using 10,000 permutations.

### Clinical features

We analyzed the association between the polymorphisms in the IL23R gene and a series of clinicopathologic features, including clinical stage, lymph node metastasis, tumor size, and the statuses of P53, ER, PR and Her-2.

Only rs1884444 in IL23R showed significant association with clinicopathologic features (Table 3). The frequency of the G allele was lower (*P* = 0.0210, OR = 0.68, 95%CI = (0.94–0.49)) in Her-2-positive cases. And our findings also indicated that the frequency of genotype TT was higher (*P*<0.0001) in patients with tumors ≤2 cm, whereas the frequencies of genotype GG and the G allele were higher in patients with tumors >5 cm (*P*<0.0001 and *P* = 0.0004, respectively). No association between IL23R haplotypes and the clinical features was found in our study.

**Table 3:** Clinical features associated with rs1884444 in IL23R gene.

Genotype/Allele	Frequency no. (%)			*P* value	Frequency no. (%)		*P* value
	TZ≤2	2<TZ≥5	TZ>5		Her-2 (+)	Her-2 (−)	
TT	110 (46.61)	93 (41.70)	0 (0.00)	<0.0001	62 (51.24)	130 (41.54)	0.0679
TG	98 (41.53)	102 (45.74)	15 (53.57)	0.3875	51 (42.15)	141 (45.05)	0.5856
GG	28 (11.86)	28 (12.56)	13 (46.43)	<0.0001	8 (6.61)	42 (13.42)	0.0464
T	318 (67.37)	288 (64.57)	15 (36.59)		175 (72.31)	401 (64.06)	
G	154 (33.33)	158 (35.43)	26 (63.41)	0.0004	67 (27.69)	225 (35.94)	0.0210

Abbreviations: TZ, tumor size; Her-2, human epidermal growth factor receptor-2.

## Discussion

Although the exact mechanisms by which IL23R modulates cancer susceptibility are still unclear, the key role of IL23R in cancer has been demonstrated by recent genetic studies in which genetic variants in the IL23R gene were associated with oral, gastric and esophageal cancer [12], [13], [14]. We hypothesized that IL23R may affect the development of breast cancer. Therefore, we investigated the influence of the IL23R gene polymorphisms in the pathogenesis of breast cancer.

To date, no studies have investigated the association between IL23R gene polymorphism and human breast cancer. In our case-control study, for the first time we investigated the association between two SNPs in IL23R gene and the risk of sporadic breast cancer in Chinese Han women. Genotyping of the two SNPs in our study showed that rs10889677, located at the 3′-UTR of the IL23R gene, was correlated with the risk of breast cancer. The homozygous AA genotype and the A allele were more frequent in breast cancer patients, whereas the C allele was associated with early age of breast cancer onset. The frequency of the haplotype T_rs1884444_A_rs10889677_ was higher in patients than in controls, whereas the frequency of the haplotype T_rs1884444_C_rs10889677_ was lower in patients than in controls. Antonie et al suggested that rs10889677 A allele could loss the binding capacity of Let-7e and Let-7f [15]. Thus it seemed logical that the AA genotype and the A allele led to the loss of a regulatory miRNA site and increased the IL23R expression, and then increased the risk of breast cancer. Interestingly however, in the case-only study we found this rs10889677 C allele effected age of onset of breast cancer in a dose-dependent manner, which might imply that the two alleles have different effects at different ages. But further larger epidemiological studies would be needed to confirm the result. Thus, our study reveals a novel SNP in the IL23R gene that may be associated with the risk and the early age of onset of sporadic breast cancer.

The frequency of the G allele in rs1884444, located at exon 2 of IL23R gene, was lower in Her-2-positive patients than in Her-2-negative patients. Hormone and Her-2 receptors are two important pharmaceutical targets that affect the survival of patients with metastatic breast cancer [17]. In addition, the humanized antibody targeting Her-2 (Trastuzumab) is used in combination with chemotherapy to treat patients with breast cancers overexpressing Her-2. These data suggested that patients with the rs1884444 G allele were less likely to be eligible to the treatment with Trastuzumab. And our findings also indicated that the frequency of genotype TT was higher in patients with tumors ≤2 cm, whereas the frequencies of genotype GG and the G allele were higher in patients with tumors >5 cm. Therefore, we speculated that the rs1884444 G allele might be a marker of poor prognosis for breast cancer patients.

IL23R constitutively associates with Janus kinase (JK) 2 and also allows binding of Signal Transducer and Activator of Transcription (STAT) 3 in a ligand-dependent manner, whose phosphorylation and consequent dimerization trigger downstream expression of genes, such as IL-17 and IL-22 [16]. The rs10889677 SNP in IL23R possibly can cause overexpression of the receptor, driving differentiation of Th1 helper T cells toward a Th17 subpopulation, resulting in an increased release of IL-17 from these cells. This would subsequently lead to the release of other cytokines (e.g. TNF), causing chronic inflammation and cancer [18], [19]. The IL-23/IL-17 axis has diverse roles in tumorigenesis. On one hand, it may promote a proinflammatory environment which is associated with cancer risk. On the other hand, the IL-23/IL-17 pathway might also lead to tumor immune surveillance of elimination, equilibrium and escape [20].

Our previous study has suggested the association between IL-17 polymorphisms and risk of breast cancer [4]. This finding will be of importance to further understand the role of the IL-23/IL-17 axis in breast cancer and could help to develop better therapies. However, further replication studies in a large cohort would be needed to confirm the suggested association.

## Materials and Methods

### Subjects

Our study included 491 Chinese women with breast cancer but no family history of cancer and 502 healthy women. The sporadic breast cancer patients were selected from the Department of Breast Surgery (The Third Affiliated Hospital of Harbin Medical University, Heilongjiang Province) and ranged in age from 27 to 91 years old (mean age 49.5±10.3 years). The breast cancer patients were diagnosed based on surgical and pathological symptoms. The control group consisted of 502 age-matched healthy Han women (mean age 49.0±9.9 years) who were volunteers in the same district and had no history of tumors or inflammatory diseases. And imaging examinations were done for the volunteers to rule out breast cancer. Both the breast cancer group and the healthy control group were from the Heilongjiang Province of China and all of the blood samples were obtained from September 2008 to May 2009. The Ethics Committee of Harbin Medical University approved the study protocol, and written informed consent was obtained from all of the participating subjects. After providing informed consent, each participant was interviewed to collect detailed information on demographic characteristics (Table S1) and provided 5 ml venous blood.

### Clinicopathological evaluation

The clinicopathologic information of breast cancer patients was shown in Table S2. We retrospectively evaluated conventional clinicopathological factors and the immunohistochemistry (IHC) results for four biological factors (ER, ZM0104; PR, ZM0215; Her-2, ZM0065; P53, ZA-0501; Beijing ZhongShan GoldenBridge Biological Technology CO., LTD, China) using paraffin embedded tissues according to the reported recommendations for tumor marker prognostic studies (REMARK) [21]. The tumor clinical stage was assessed according to the criteria described in the International Union Against Cancer (UICC).

A cut-off value of 10% of positively stained nuclei was used to define ER and PR positivity; Her-2 was scored as 0–3+ according to membrane staining. Cases with IHC scores of 3+ or 2+ with gene amplification by fluorescence in situ hybridization (FISH) were considered positive for Her-2. Cells with positive staining for P53 were counted and expressed as a percentage. We scored the immunostained slides as 0–3+ (0, negative; 1+, ≤25%; 2+, 25–50%; 3+, >50%). For the prognosis comparison, low expression was defined as P53 ≤25% (median values for all evaluated tumors).

### DNA extraction

Genomic DNA was isolated from EDTA anti-coagulated whole blood using the AxyPrep Blood Genomic DNA Miniprep Kit (Axygen Biotechnology, USA). Each DNA sample was stored at −20°C until analysis.

### SNP selection and genotyping

Tag SNP selection performed with Haploview v4.0 through pairwise tagging (r^2^>0.80) and minor allele frequencies >0.1 using Chinese (CHB) genotype data from HapMap (http://www.hapmap.org/) covering the IL23R gene. Two SNPs (rs10889677 located at the 3′-UTR and rs1884444 located at codon 3 in exon 2) of the IL23R gene were selected as tags.

The SNaPshot SNP assay was performed to detect the dimorphism at the two SNP loci. The PCR primer pairs used to amplify the DNA sequences are as follows: rs10889677 (F: 5′-CGGGAGCTCCATGCCTTTTTA-3, R: 5′-TGAGGCGTCCACATAATGCTGTT-3′) and rs1884444 (F: 5′-TCCCTAATCAAAGGTTCCCATCAA-3′, R: 5′- CCTCCATGACACCAGCTGAAGA-3′). PCR was performed in a 20 µl reaction mixture containing 1 µl (10 ng) of template DNA, 1 µM of each primer, 0.3 mM of each deoxynucleotide triphosphate, 3.0 mM of MgCl_2_, and 1 U HotStarTaq polymerase (Qiagen Inc., USA) with 1× HotStarTaq buffer. The PCR program consisted of an initial melting step of 15 minutes at 95°C; 11 cycles of 20 seconds at 94°C, 40 seconds at 65°C-0.5°C/cycle, and 90 seconds at 72°C; 24 cycles of 20 seconds at 94°C, 30 seconds at 59°C, and 90 seconds at 72°C; and a final elongation step of 2 minutes at 72°C. To purify the PCR products, 1 U SAP and 1 U Exonuclease I were mixed with 10 µl PCR product for 1 hour at 37°C and 15 minutes at 75°C. The SNaPshot multiplex single-base extension reaction primer sequences of each SNPs are as follows: rs10889677 (SF: TTTTTTTTTTTTTTTTTTTTTTTTTTTTTTTTTTTTTTTAATTTTAGCCATTCTTCTGCCT) and rs1884444 (SR: TTTTTTTTTTTTTTTTTTTTTTTTTTTTTTTTTTACTGCATCCCATTGAATAGTGAC). The extension reaction was performed in a 10 µl reaction mixture containing 5 µl of the SNaPshot Multiplex Kit (Applied Biosystems, USA), 2 µl of purified PCR products, 0.8 µM of the extension reaction primer, and 2 µl water. The PCR program was 1 minute at 96°C; 28 cycles of 10 seconds at 96°C, 5 seconds at 50°C, and 30 seconds at 60°C; and 4°C as the holding temperature. Finally, 10 µl of the extension product was purified with 1 U SAP for 1 hour at 37°C and inactivated for 15 minutes at 75°C.

The resulting data were analyzed with an ABI3130XL sequencer and GeneMapper™ 4.0 Software (Applied Biosystems, Co. Ltd., USA). The SNP genotyping figure is shown in Figure S1. To ensure quality control (QC), genotyping was performed by researchers blinded to the case/control status of the subjects, and a random sample of 5% of the cases and controls was genotyped twice by different persons, with a reproducibility of 100%.

### Statistical analysis

The Hardy-Weinberg equilibrium (HWE) was tested by chi-square test to compare the expected genotype frequencies with observed genotype frequencies in breast cancer cases and cancer-free controls. The frequencies of alleles and genotypes were obtained through direct counting. The comparisons of the distributions of the genotype, allele and haplotype frequencies were performed using the Chi-square test. The relative risk associated with rare alleles, genotypes and haplotypes was estimated as an odds ratio (OR) with a 95% confidence interval (CI). ANOVA tests were used for analyzing the age of onset in breast cancer patients by IL23R genotypes. We used Haploview software to construct haplotypes and estimate haplotype frequencies for both cases and controls. Only the haplotypes that occurred at a frequency of ≥10% in the case-control population were selected in our study. To obtain a measure of significance that was corrected for multiple testing bias, we ran 10,000 permutations to compute *P*-values using the Haploview program. All statistical tests were two-sided, and the statistical significance was defined as *P*<0.05.

## Supporting Information

Figure S1 SNP genotyping figures of rs10889677 and rs1884444 in IL23R gene.
(TIF)

Table S1 Frequency distribution of selected variables in breast cancer cases and cancer-free controls.
(DOC)

Table S2 Clinicopathologic description of the breast cancer patients.
(DOC)
