# Supplementary figures and images for: A miRNA Binding Site Single-Nucleotide Polymorphism in the 3′-UTR Region of the IL23R Gene Is Associated with Breast Cancer
Source: PLoS One. 2012 Dec 11;7(12):e49823. doi: 10.1371/journal.pone.0049823 (PMC3519811; doi:10.1371/journal.pone.0049823)

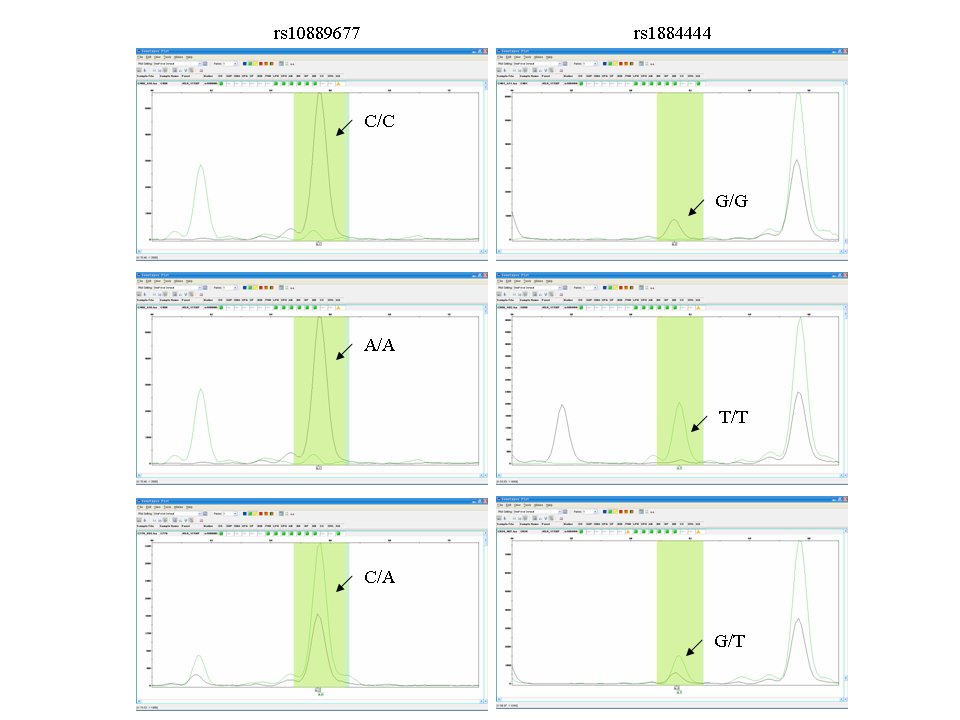

Supplement: Figure S1 — SNP genotyping figures of rs10889677 and rs1884444 in IL23R gene. (TIF) [file pone.0049823.s001.tif]
