# Supplementary material for: A miRNA Binding Site Single-Nucleotide Polymorphism in the 3′-UTR Region of the IL23R Gene Is Associated with Breast Cancer
Source: PLoS One. 2012 Dec 11;7(12):e49823. doi: 10.1371/journal.pone.0049823 (PMC3519811; doi:10.1371/journal.pone.0049823)
Supplement: Table S1 — Frequency distribution of selected variables in breast cancer cases and cancer-free controls. (DOC) [file pone.0049823.s002.doc]

**Table S1 Frequency distribution of selected variables in breast cancer cases and cancer-free controls**

| Variables | No. of subjects (%) | | *P* |
| --- | --- | --- | --- |
|  | Cases (n=491) | Controls (n=502) |  |
| Age (y) |  |  | 0.6865 |
| ≤50 | 284 (57.84) | 284 (56.57) |  |
| >50 | 207 (42.16) | 218 (43.43) |  |
| BMI (kg/m^2^) |  |  | <0.0001 |
| <18 | 13 (2.65) | 13 (2.59) |  |
| 18-25 | 287 (58.45) | 377 (75.10) |  |
| >25 | 187 (38.09) | 112 (22.31) |  |
| No. of parity |  |  | 0.0006 |
| 0 | 24 (4.89) | 30 (5.98) |  |
| 1 | 281 (57.23) | 339 (67.53) |  |
| ≥2 | 186 (37.88) | 133 (26.49) |  |
| No. of abortion |  |  | <0.0001 |
| 0 | 234 (47.66) | 151 (30.08) |  |
| 1 | 137 (27.90) | 182 (36.25) |  |
| ≥2 | 120 (24.44) | 169 (33.67) |  |
| Duration of breast feeding (mo) |  |  | <0.0001 |
| ≤12 | 250 (50.92) | 340 (67.73) |  |
| >12 | 241 (49.08) | 162 (32.27) |  |
| Age at menarche |  |  | 0.0117 |
| ≤15 | 255 (51.93) | 300 (59.76) |  |
| >15 | 236 (48.07) | 201 (40.04) |  |
| Menopause |  |  | 0.7879 |
| No | 279 (56.82) | 281 (55.98) |  |
| Yes | 212 (43.18) | 221 (44.02) |  |
| Family history of any cancer |  |  | 0.0227 |
| No | 389 (79.23) | 433 (86.25) |  |
| Yes | 92 (18.74) | 69 (13.75) |  |
