# Supplementary material for: A miRNA Binding Site Single-Nucleotide Polymorphism in the 3′-UTR Region of the IL23R Gene Is Associated with Breast Cancer
Source: PLoS One. 2012 Dec 11;7(12):e49823. doi: 10.1371/journal.pone.0049823 (PMC3519811; doi:10.1371/journal.pone.0049823)
Supplement: Table S2 — Clinicopathologic description of the breast cancer patients. (DOC) [file pone.0049823.s003.doc]

**Table S2** Clinicopathologic description of the breast cancer patients (*n*=491)

| Clinicopathologic information | Case no. (%) |
| --- | --- |
| Histology |  |
| Infiltrative ductal carcinoma | 420 (85.54) |
| Intraductal carcinoma | 36 (7.33) |
| Mucinous adenocarcinoma | 13 (2.65) |
| Infiltrative lobular carcinoma | 12 (2.44) |
| Medullary carcinoma | 4 (0.81) |
| Others | 6 (1.22) |
| Clinical Stage (UICC) |  |
| 0 | 2 (0.41) |
| Ⅰ | 124 (25.25) |
| Ⅱ | 256 (52.14) |
| Ⅲ | 37 (7.54) |
| Ⅳ | 8 (1.63) |
| Unknown | 64 (13.03) |
| Tumor size (cm) |  |
| TZ≤2 | 236 (48.07) |
| 2<TZ≤5 | 223 (45.42) |
| TZ>5 | 28 (5.70) |
| Unknown | 4 (0.81) |
| LN involvement |  |
| Positive | 204 (41.55) |
| Negative | 261 (53.15) |
| Unknown | 26 (5.30) |
| P53 |  |
| Positive | 98 (19.96) |
| Negative | 335 (68.23) |
| Unknown | 58 (11.81) |
| ER |  |
| Positive | 266 (54.18) |
| Negative | 169 (34.42) |
| Unknown | 56 (11.40) |
| PR |  |
| Positive | 237 (48.27) |
| Negative | 199 (40.53) |
| Unknown | 55 (11.20) |
| Her-2 |  |
| Positive | 121 (24.64) |
| Negative | 313 (63.75) |
| Unknown | 57 (11.61) |

**Abbreviations：**

LN, lymph node; P53, tumor protein 53; ER, estrogen receptor; PR, progesterone receptor; TZ, tumor size; Her-2, human epidermal growth factor receptor-2
